# Sensitivity and Specificity of Cardiac Tissue Discrimination Using Fiber-Optics Confocal Microscopy

**DOI:** 10.1371/journal.pone.0147667

**Published:** 2016-01-25

**Authors:** Chao Huang, Frank B. Sachse, Robert W. Hitchcock, Aditya K. Kaza

**Affiliations:** 1 Department of Bioengineering, University of Utah, Salt Lake City, Utah, United States of America; 2 Nora Eccles Harrison Cardiovascular Research and Training Institute, University of Utah, Salt Lake City, Utah, United States of America; 3 Department of Cardiac Surgery, Boston Children’s Hospital and Harvard Medical School, Boston, Massachusetts, United States of America; Harbin Medical University, CHINA

## Abstract

Disturbances of the cardiac conduction system constitute a major risk after surgical repair of complex cases of congenital heart disease. Intraoperative identification of the conduction system may reduce the incidence of these disturbances. We previously developed an approach to identify cardiac tissue types using fiber-optics confocal microscopy and extracellular fluorophores. Here, we applied this approach to investigate sensitivity and specificity of human and automated classification in discriminating images of atrial working myocardium and specialized tissue of the conduction system. Two-dimensional image sequences from atrial working myocardium and nodal tissue of isolated perfused rodent hearts were acquired using a fiber-optics confocal microscope (Leica FCM1000). We compared two methods for local application of extracellular fluorophores: topical via pipette and with a dye carrier. Eight blinded examiners evaluated 162 randomly selected images of atrial working myocardium (n = 81) and nodal tissue (n = 81). In addition, we evaluated the images using automated classification. Blinded examiners achieved a sensitivity and specificity of 99.2±0.3% and 98.0±0.7%, respectively, with the dye carrier method of dye application. Sensitivity and specificity was similar for dye application via a pipette (99.2±0.3% and 94.0±2.4%, respectively). Sensitivity and specificity for automated methods of tissue discrimination were similarly high. Human and automated classification achieved high sensitivity and specificity in discriminating atrial working myocardium and nodal tissue. We suggest that our findings facilitate clinical translation of fiber-optics confocal microscopy as an intraoperative imaging modality to reduce the incidence of conduction disturbances during surgical correction of congenital heart disease.

## Introduction

Congenital heart disease is the most common birth defect, which affects approximately 1% of all live births [1]. The main treatment modality is surgical repair. Tremendous progress was made over the past half century to lower the incidence of conduction disturbances following surgical repair of congenital heart defects. The primary therapeutic intervention in treating persistent conduction disturbances is permanent pacemaker implantation. Improved understanding of the anatomy of the conduction system and advances in surgical techniques have reduced the incidence of disturbances after repair of some common congenital lesions such as ventricular septal defect from greater than 10% to 1–3% [2–4]. However, repair of complex cardiac malformations including abnormally related great arteries, atrioventricular discordance and single ventricle physiology are still associated with high incidence of permanent pacemaker implantation. For instance, the incidence is 41–45% after repair of congenitally corrected transposition of the great arteries [5, 6].

Currently, in order to prevent conduction disturbances, surgeons rely on anatomical landmarks to approximate the location of the cardiac conduction system. In complex cardiac malformations where the location of these specialized tissues is distorted, the established approach may not be able to localize the conduction tissue [7, 8]. With an increasing prevalence of complex cardiac malformations [9], and therefore increasing occurrence of surgical correction for these less common malformations, a more reliable and accurate intraoperative method for localizing the conduction system is indicated. There have been previous attempts at electrophysiological identification of the conduction system during open heart surgery [8, 10]. However, the reliance on atrial pacing to achieve reliable recordings without arrhythmias limited the application of these electrophysiology-based localization techniques.

Fiber-optics confocal microscopy (FCM) allows for real-time imaging of cellular and sub-cellular features up to 100 μm below the specimen’s surface. Current clinical applications of FCM include imaging of tissues in gastrointestinal [11], respiratory [12] and urinary [13] system. We recently introduced approaches based on FCM and local delivery of extracellular fluorophores for imaging in the living heart [14, 15]. In these studies we demonstrated feasibility of these approaches for cardiac tissue discrimination in the living heart of a rodent model, as well as in the fixed human fetal and pediatric heart. In particular, we were able to discriminate the specialized tissue of the conduction system in the sinoatrial node (SAN) and atrioventricular node (AVN) from atrial working myocardium (AWM) based on the spatial arrangement of the extracellular space. We found that AWM, which consists of highly aligned myocytes, presented extracellular space that had a regular striated arrangement. In contrast, nodal tissue presented an irregular reticular arrangement of the extracellular space. Furthermore, in these studies we evaluated several extracellular fluorophores, as well as local and systemic approaches for delivery of these fluorophores. Our studies suggest that AWM and nodal tissue can be identified based on their characteristic microstructural arrangement regardless of the abovementioned fluorophores or delivery approach.

In this study, we investigated the performance of human and automated classification systems in discriminating images of AWM and specialized tissue of the conduction system acquired. We assessed the performance of these classification systems based on measures of sensitivity and specificity [16]. We defined sensitivity, which is the true positive rate of a classification system, as the proportion of AWM images correctly identified as such. Similarly, we defined specificity, which describes the true negative rate, as the proportion of specialized tissue images correctly identified as specialized tissue of the conduction system. Our hypothesis is that human and automated tissue classification can discriminate FCM images of AWM and specialized tissue of the conduction system with high sensitivity and specificity. For this purpose, we used conventional three-dimensional laser-scanning confocal microscopy, FCM, and two methods for local delivery of extracellular fluorophores to acquire images from AWM and nodal tissue from the SAN and AVN. Spatial regularity of the extracellular space in the images of AWM and nodal tissue was measured using texture analysis. We performed receiver operating characteristic (ROC) analysis to compare sensitivity versus specificity across a range of spatial regularity thresholds measured over three image sets [17]. From the ROC analysis of the distributions of spatial regularity, we determined optimal cutoff values used in automated tissue classification. Subsequently, we evaluated the sensitivity and specificity of blinded human examiners, as well as automated classification based on optimal cutoff values in discriminating a set of randomly selected images of AWM and nodal tissue.

## Materials and Methods

### Heart Tissue Preparations

Animal procedures were approved by the University of Utah Institutional Care and Use Committee and followed the guidelines of the National Institutes of Health Guide for the Care and Use of Laboratory Animals. Human studies were reviewed and approved for exemption by the institutional review board at the University of Utah. Details on the preparation and fluorescent labeling of fixed tissue samples from rodent, neonatal lamb, and human hearts are provided in the S1 Appendix. In particular, fixed tissue samples were fluorescently labeled with wheat germ agglutinin (WGA) to visualize constituents of the extracellular space, the outer membrane of cells and the tissue microstructure [18, 19]. Furthermore, fixed tissue samples from rodent were also immunolabeled with antihyperpolarization-actived cyclic nucleotide-gated potassium channel 4 (HCN4) to detect cells of the conduction system [20, 21].

### Imaging of Fluorescently Labeled Fixed Tissue

**Fig 1:**
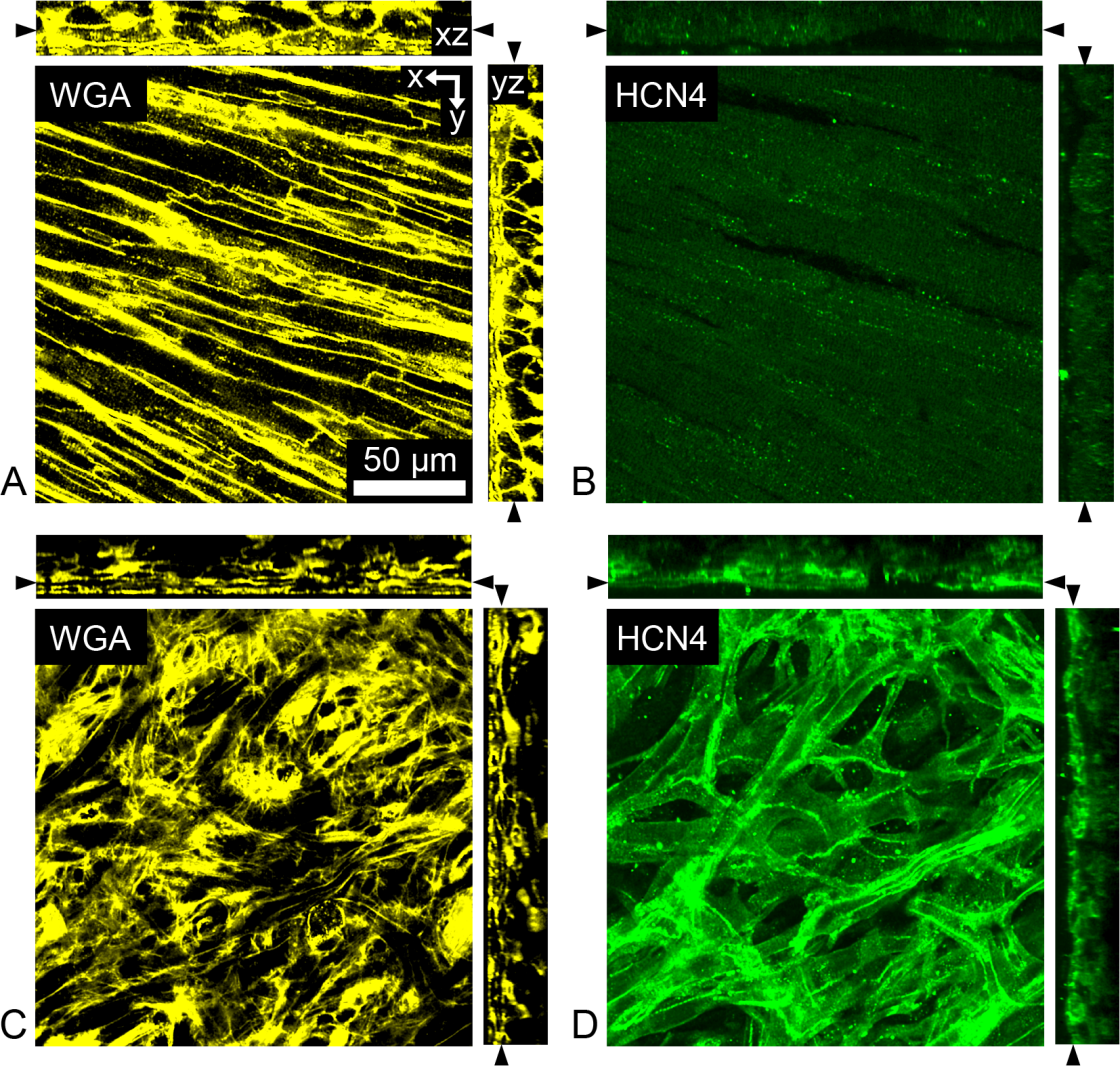
Laser-scanning confocal images of fixed rodent tissue preparations. Cross-section through image stacks of (A,B) atrial working myocardium and (C,D) sinoatrial node tissue. Preparations were fluorescently labeled with (A,C) wheat germ agglutinin to visualize constituents of the extracellular space, the outer membrane of cells and the tissue microstructure. Preparations were also immunofluorescently labeled with (B,D) anti-hyperpolarization-activated cyclic nucleotide-gated potassium channel 4 to detect cells of the conduction system.

Fluorescently labeled fixed tissue preparations from rodent, neonatal lamb, and human were imaged using conventional confocal microscopy based on established methods [14, 22] and outlined in the S1 Appendix. Representative cross-sections through image stacks from fixed rodent tissue preparations are presented in Fig 1. These image stacks were acquired from the epicardial surface into the subepicardial AWM (Fig 1A and 1B) and SAN (Fig 1C and 1D) tissue labeled with WGA (Fig 1A and 1C) and anti-HCN4 (Fig 1B and 1D). All acquired high magnification image stacks using conventional confocal microscopy were indexed based on anatomical origin of the imaged tissue region, i.e. AWM or nodal tissue and stored. A particular set of images (referred to as CCM images) from indexed image stacks acquired using conventional confocal microscopy of WGA-labeled rodent tissues were stored for subsequent image analysis. Additionally, we acquired anatomical overviews of the right atrium from fixed rodent hearts labeled with WGA and anti-HCN4. A representative anatomical overview of the HCN4 distribution in the partial right atrium of rodent is presented in S1 Fig.

### FCM Imaging of the Living Heart

In addition to conventional confocal microscopy of fixed tissue preparations, we performed FCM on Langendorff-perfused arrested hearts from rodent and neonatal lamb. Our experimental conditions reproduced cardioplegic arrest commonly required for pediatric heart surgery. Two-dimensional image sequences were acquired using a FCM system (FCM 1000; Leica Microsystems GmbH, Wetzlar, Germany) and two methods for local delivery of fluorescent dye [14, 15].

The first method was based on topical application of fluorescent dye via pipette to AWM, SAN and AVN tissue regions of perfused rodent and neonatal lamb hearts. The fluorescent dye solution consisted of 3 or 10 kDa dextran conjugated to Alexa Fluor 488 (Invitrogen, Carlsbad, CA, 1:8) dissolved in Tyrode’s solution to a final concentration of 125 μg/mL. Following dye delivery, tissue regions were imaged using the FCM system equipped with a custom fiber-optics microprobe having a numeric aperture of 0.8 (UltraMiniO; Mauna Kea Technologies, Paris, France). Two-dimensional image sequences were acquired at a lateral resolution (*xy* dimensions) of 1.8 μm, optical sectioning (*z*) of 10 μm, *xy* field of view of 169 by 120 μm, frame rate of 12 Hz, and z-scan range of 50 μm. Images from rodent acquired with this topical method of dye delivery are subsequently referred to as FCM_topical_ images

Rodent hearts were also imaged using FCM and a method of local dye delivery based on a dye carrier affixed to the tip of the FCM imaging microprobe. Dye carriers were fabricated according to a recently described method [15] and loaded for 15 min with fluorescent dye, sodium fluorescein [Fluorescite® (fluorescein injection, USP) 10%; Alcon, Fort Worth, TX, US; 1:1000]. Images from AWM, SAN, and AVN regions of perfused rodent hearts were acquired using this method with a custom fiber-optics microprobe having a numeric aperture of 0.8 (UltraMiniOWD30; Mauna Kea Technologies) at a lateral resolution (*xy*) of 1.4 μm, optical sectioning (*z*) of 7 μm, *xy* field of view of 186 by 130 μm, frame rate of 12 Hz, and a z-scan range of 26 μm. Images from rodent acquired with the dye carrier method are subsequently referred to as FCM_carrier_ images. FCM_topical_ and FCM_carrier_ images were indexed based on anatomical origin, i.e. AWM or nodal, and stored for subsequent image analysis.

### Image Analysis

We characterized the spatial regularity of the extracellular space in CCM, FCM_topical_, and FCM_carrier_ images using two methods of image texture analysis. A detailed description of the characterization is provided in the S1 Appendix. The characterization allowed us to quantitatively measure the spatial regularity, denoted as *I*_*15*_, in all CCM, FCM_topical_, and FCM_carrier_ images using both methods of texture analysis. We subsequently developed automated classification systems based on the *I*_*15*_ distribution of these image sets. In our classification scheme we defined true positive and true negative outcomes as AWM correctly classified as AWM and nodal images correctly classified as nodal, respectively.

#### Automated classification of tissue images

We evaluated automated methods for classification of AWM and nodal images based on texture analysis. We mapped *I*_*15*_ values calculated from both Fourier and second order moment analyses of CCM, FCM_topical_, and FCM_carrier_ images to ROC curves. ROC curves were obtained using the “perfcurve” function of the MATLAB (The Mathworks Inc, Natick, MA) Statistics Toolbox. In addition, for each ROC curve, we calculated an optimal cutoff value, which maximizes the product of sensitivity and specificity and therefore minimizes both the false-positives and false-negative cases. In calculation of the optimal cutoff values, additional weight was given for the misclassification of negative results. We defined false positives (i.e., nodal misclassified as AWM) two times as costly as false negatives (i.e., AWM misclassified as nodal). Eight-one images from CCM, FCM_topical_, and FCM_carrier_ were randomly selected and classified as either AWM or nodal images using automated methods based on these optimal cutoff values. The sensitivity and specificity of these automated methods in discriminating these cardiac tissue types was determined from these classifications.

#### Human examiner classification of tissue images

Eight human examiners reviewed the set of CCM, FCM_topical_, and FCM_carrier_ images previously classified by automated methods. The examiners were asked to classify the images as AWM or nodal following a 5 min training phase. Training consisted of a slide presentation of previously indexed images of AWM and nodal tissue that illustrated microstructural features indicative of each tissue type. Examiners were blinded to the classification of the images. Sensitivity and specificity of these examiners in discriminating AWM and nodal images was determined from these classifications. The previously defined classification scheme for a true positive and true negative outcome was used in determining the sensitivity and specificity.

## Results

### Microscopic Imaging of Cardiac Tissue Microstructure

**Fig 2:**
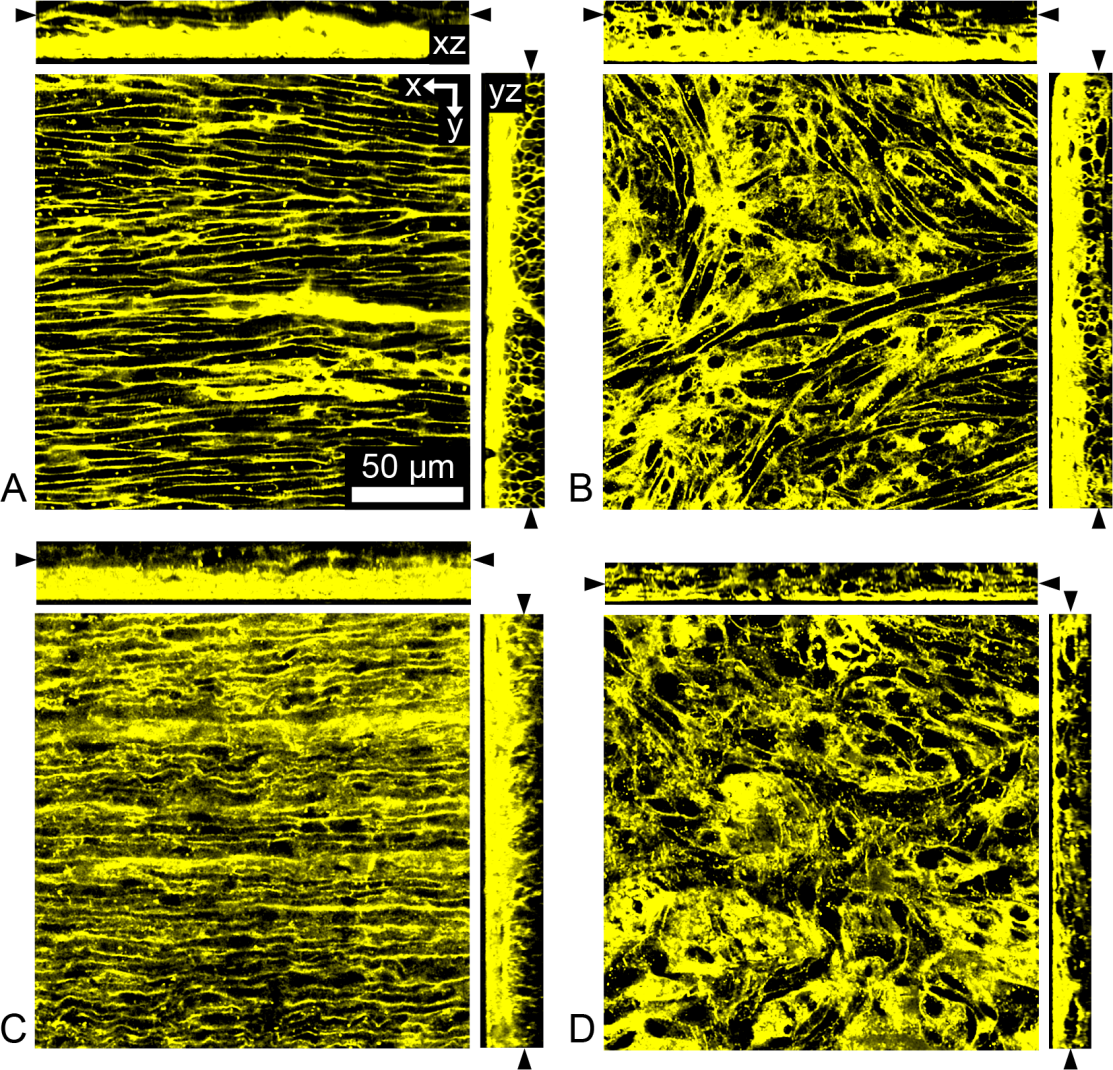
Laser-scanning confocal images of neonatal ovine and human tissue preparations. Cross-section through image stacks of fixed (A,B) neonatal lamb and (C,D) human tissue preparations labeled with wheat germ agglutinin. (A,C) Image stacks acquired from the epicardial surface into the subepicardial atrial working myocardium. (B,D) Image stacks acquired from the endocardial surface into the subendocardial atrioventricular node tissue.

The neonatal lamb is a common animal model used to study pediatric cardiac diseases. We thus studied the microstructural arrangement of the AWM and nodal tissue in this model. Conventional confocal microscopic imaging of fixed neonatal lamb preparations labeled with WGA are presented in Fig 2A and 2B. The anatomical location of the SAN and AVN in this species is grossly similar as in other mammals including rodent and human. The microstructure of the extracellular space within AWM in neonatal lamb was characterized by a regular striated arrangement (Fig 2A). In contrast, the microstructure of the extracellular space found within the AVN was characterized by a heterogeneous, irregular arrangement (Fig 2B). The same microstructural features have been previously observed by us in both rodent and human [14]. For comparison, we present example cross-sections through image stacks of fixed human tissue in Fig 2C and 2D.

**Fig 3:**
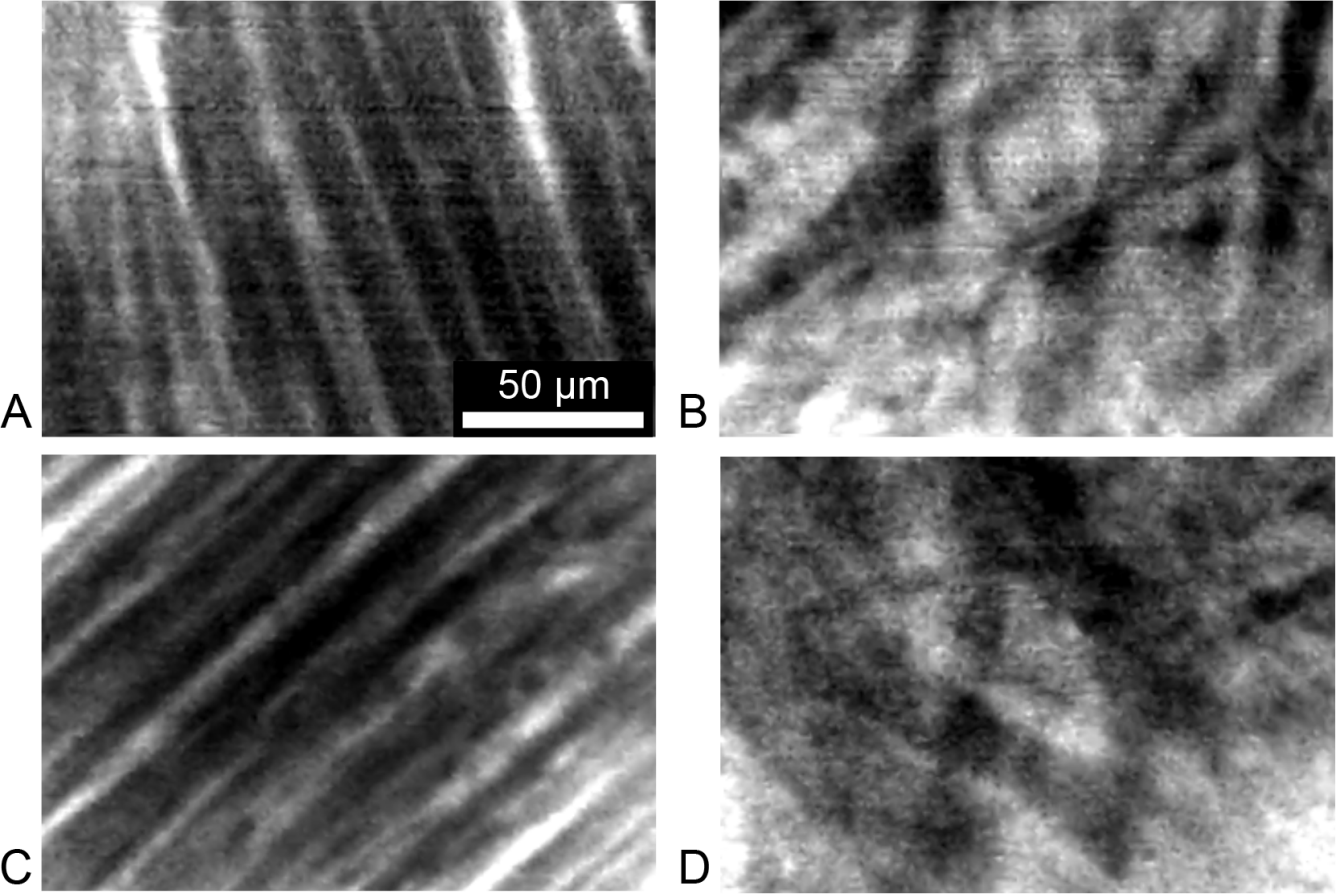
Fiber-optics confocal microscopy of the living arrested heart. Example fiber-optics confocal microscopy images from (A,B) rodent and (C,D) neonatal lamb. Images were acquired using a UltraMiniO imaging microprobe after topical administration of dextran-Alexa Fluor 488 via pipette to a region of (A,C) atrial working myocardium and (B,D) sinoatrial node. Note that the applied fiber-optics confocal microscope yields images of lower spatial resolution than state-of-the-art laser-scanning confocal microscopes (Fig 1).

In Fig 3 we present FCM images from rodent (Fig 3A and 3B) and neonatal lamb (Fig 3C and 3D) of AWM (Fig 3A and 3C) and AVN (Fig 3B and 3D) acquired after topical application of dextran-Alexa Fluor 488. Similar microstructural features are apparent in these images as described above for conventional confocal microscopy.

### Analyses of Cardiac Tissue Microstructure from FCM Images

**Fig 4:**
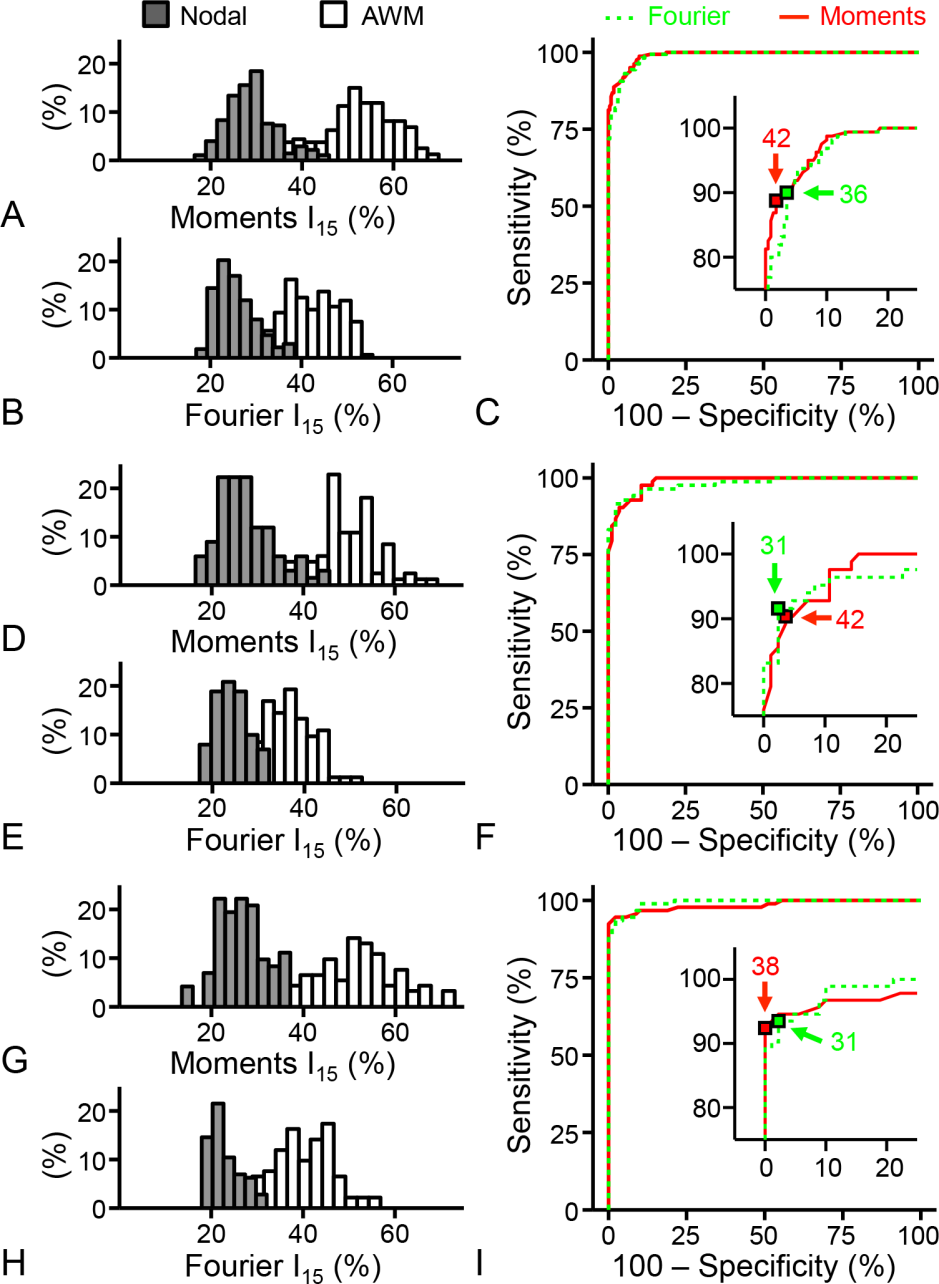
Receiver operating characteristic analysis of indexed image sets. Histograms and receiver operating characteristic curves of *I*_*15*_ for (A-C) CCM, (D-F) FCM_topical_ and (G-I) FCM_carrier_ images based on Fourier (green dotted line) and image moment (red solid line) texture analysis. A bimodal *I*_*15*_ distribution was observed with indexed nodal (filled histogram) and atrial working myocardium (AWM, unfilled histogram) images grouped around lower and higher maxima, respectively. Inset within ROC curves show a magnified view of the curve closest to the region of perfect classification (upper left corner). Optimal cut-off values from Fourier (green square) and image moment (red square) texture analysis of the (C) CCM, (F) FCM_topical_ and (I) FCM_carrier_ images are shown in the insets.

Image stacks and sequences of AWM, SAN and AVN regions from rodent were acquired using conventional confocal microscopy, FCM and two methods for fluorescent dye delivery. The resulting images, CCM, FCM_topical_, and FCM_carrier_, were indexed based on anatomical origin of imaged region, stored (see Data Availability Statement) and subsequently analyzed using texture analysis. In our analysis, we extracted features of the microstructural arrangement from the extracellular space within these images, in particular the spatial regularity. We then mapped the spatial regularity to a simple score denoted as *I*_*15*_. Analysis of ROC curves generated from these *I*_*15*_ values allowed us to determine optimal cutoff values for automated classification. In Fig 4 we present histograms and ROC curves of *I*_*15*_ for CCM (Fig 4A–4C), FCM_topical_ (Fig 4D–4F), and FCM_carrier_ (Fig 4G–4I) images for both Fourier and image moment based texture analysis. We observed a characteristic bimodal distribution of *I*_*15*_ regardless of the method of texture analysis or imaging approach. The distributions coincided distinctively with indexed anatomical regions. *I*_*15*_ values derived from nodal and AWM images were distributed around the lower and higher maxima, respectively. The ROC curves were obtained by varying the decision threshold between the minimum and the maximum *I*_*15*_ of CCM (Fig 4C), FCM_topical_ (Fig 4F), and FCM_carrier_ (Fig 4I) images calculated from Fourier (green dotted line) and image moment (solid red line) analysis. The inset within the ROC curves shows a magnified view of the curve closest to the region of perfect classification. The inset also shows optimal cutoff values for CCM, FCM_topical_, and FCM_carrier_ images based on Fourier and image moment analysis. The optimal cutoff value was calculated based on minimizing the distance between the ROC curve and the upper left corner of the plot with the addition of a weighting factor based on the misclassification cost of AWM and nodal images. Details on the ROC analysis are presented in S1 Table, including sample sizes as well as the sensitivity and specificity for each optimal cutoff value.

### Evaluation of Human and Automated Classification

In a last set of experiments we evaluated the performance of human and automated classification systems in discriminating AWM and nodal images. A random set of 81 AWM and 81 nodal images from each of the CCM, FCM_topical_, and FCM_carrier_ image sets were evaluated by 8 human examiners. Preceding the evaluation, the examiners were trained to identify hallmark features in the microstructural arrangement of the extracellular space that allowed for AWM and nodal identification. In addition, the same set of images was evaluated by automated classification systems based on optimal cutoff values from ROC curves and two methods of image texture analysis described above. The sensitivity and specificity of human and automated classification systems in discriminating AWM and nodal images are shown in Table 1. Human examiner results are presented as mean ± standard error. We achieved a sensitivity of 99.2% ± 0.3 and specificity of 98.0% ± 0.7 in human examiner evaluation of FCM_carrier_ images. FCM_carrier_ images were acquired using the dye carrier method of fluorescent dye delivery. Approximately 1 AWM image was misclassified as nodal (false negative) and approximately 2 nodal images were misclassified as AWM (false positive) from the 81 AWM and 81 nodal images classified. In comparison, human examiners misclassified approximately 1 AWM (99.2% ± 0.3 sensitivity) and 5 nodal (94.0% ± 2.4 specificity) images in evaluating images acquired using dye application via pipette (FCM_topical_). A comprehensive summary of the sensitivity and specificity for each human examiner and each image set evaluated is presented in S2 Table. Sensitivity and specificity for the automated method of classification based on Fourier analysis was 97.5% and 95.1%, respectively for the FCM_carrier_ images. In this case, approximately 2 AWM and 4 nodal images were misclassified from the total 162 FCM_carrier_ images. In comparison, sensitivity and specificity of automated classification based on image moments was 100% (0 AWM images misclassified) and 92.6% (6 nodal images misclassified), respectively.

**Table 1:** Summary of Human and Automated Classification of Confocal Images.

	Examiner (n = 8)		Fourier		Moments	
	SEN	SPE	SEN	SPE	SEN	SPE
Images	% ± SE		%		%	
CCM	100.0 ± 0.0	87.5 ± 3.3	100.0	95.1	100.0	88.9
FCM_topical_	99.2 ± 0.3	94.0 ± 2.4	98.8	91.4	98.8	91.4
FCM_carrier_	99.2 ± 0.3	98.0 ± 0.7	97.5	95.1	100.0	92.6

CCM, conventional confocal microscopy; FCM, fiber-optics confocal microscopy; SE, standard error; SEN, sensitivity; SPE, specificity.

## Discussion

Our studies provide evidence that human examiners can discriminate images of AWM and nodal tissue acquired using FCM with extremely high sensitivity and specificity. We also found that automated classification systems were similarly effective at discriminating these cardiac tissue types as human examiners. These results suggest that automated classification has potential to support intraoperative FCM discrimination of AWM and nodal tissue. Furthermore, the sensitivity and specificity of both human and automated classification were similar in discriminating CCM, FCM_topical_, and FCM_carrier_ (Table 1). This suggests that differences in the imaging approach such as method of fluorescent dye delivery, imaging modality, type of fluorescent or optical properties of the imaging probe have only marginal effects on the reliability of human and automated tissue discrimination. However, human examiners achieved the highest specificity in discriminating FCM_carrier_ images resulting in only approximately 2 misclassifications of nodal images as AWM images compared to approximately 4–11 nodal misclassifications by the other classifications performed (Table 1). This result is important in the context of pediatric open heart surgery as we are biased towards reducing the number of nodal rather than AWM misclassifications.

We showed previously that the approach based on sodium fluorescein and dye delivery via carrier is more appropriate for clinical applications of FCM, in particular for translation of FCM for pediatric open heart surgery [15]. This approach was used to acquire FCM_carrier_ images. In contrast to dextran-Alexa Four 488, the toxicity of sodium fluorescein has been extensively studied [23–26]. In particular, sodium fluorescein is a Food and Drug Administration class IIa drug and approved for use in ophthalmic angiography. We also demonstrated that the local dye delivery via carrier was as effective as established systemic dye delivery in visualization of the microstructural arrangement of tissue. However, local dye delivery using a dye carrier required a lower amount of dye than systemic delivery. To obtain FCM images of sufficient quality over an 8 min imaging session required approximately 4.1 and 240 μg of fluorescent dye using local and systemic dye delivery methods, respectively. The lower amount of dye required would make local dye delivery more preferable than systemic methods in the clinical environment.

Our study revealed that the microstructural features of AWM and nodal tissue in neonatal lamb resemble that of rodent and human. A characteristic microstructural feature of AWM in rodent (Figs 1A and 3A), neonatal lamb (Figs 2A and 3C) and human (Fig 2C) is the regularly striated arrangement of the extracellular space. This arrangement is a hallmark of the underlying oriented arrangement of myocytes [27]. In contrast, nodal tissue images from rodent (Figs 1C and 3B), neonatal lamb (Figs 2B and 3D) and human (Fig 2D) were characterized by an irregular, reticular arrangement. We showed previously that an irregular, reticular arrangement is a characteristic microstructural feature of nodal tissue [14]. Our findings in this study support that the microstructural arrangement of AWM and nodal tissue are similar across rodent, human and neonatal lamb. We also found that nodal tissue is located at a depth beneath the tissue surface, which is accessible to FCM imaging. Based on these findings, we suggest that neonatal lamb is an appropriate animal model for investigating the ability of FCM to delineate the conduction system, in particular for applications in pediatric open heart surgery.

### Limitations

There are certain limitations associated with FCM such as penetration depth, spatial resolution and its reliance on fluorescent dyes. Current FCM systems can image to a depth of approximately 100 μm at submicrometer resolution. We have shown that up to 50 μm in depth, microstructural features of cardiac tissue types can be discerned to allow for their discrimination, particularly in fetal and infant human hearts (Fig 2C and 2D) [14]. Therefore, we expect that the penetration depth and resolution are sufficient for clinical application of FCM for cardiac tissue discrimination, specifically for pediatric open heart surgery. In addition, recently developed *in vivo* fluorescence imaging systems work at near-infrared wavelengths. These wavelengths will allow higher penetration depth than current imaging systems using wavelengths of visible light [28]. Furthermore, *in vivo* imaging systems based on second-harmonic generation and multi-photon microscopy are rapidly evolving [29, 30]. Beyond increased depth penetration some of these systems do not require application of fluorophores. However, imaging with these systems has only been evaluated in isolated living cells or fixed tissue slices [31–33]. Evaluations in living cardiac tissue using these imaging systems have not yet been performed. However, we note that local dye delivery approaches and automated tissue discrimination methods described in this study can be easily be adapted for emerging *in vivo* imaging technologies.

We investigated automated tissue discrimination methods, which were based on *post hoc* analysis of imaged data. A clinical application of these methods would require processing in near real-time, preferably as the imaging data is being displayed. We believe that real-time automated discrimination is feasible through a combination of parallel processing, algorithm optimization, and graphics processing units. Medical imaging applications, which are computationally intensive, have seen dramatic improvements in performance by applying these technologies [34].

A further limitation is associated with the use of FCM images acquired from isolated rodent hearts in the evaluation of human and automated tissue discrimination. We expect based on the analysis of the microstructural features in rodent and human performed previously [14] and in this study, that a similarly high sensitivity and specificity of tissue discrimination can be achieved in the neonatal and infant human heart *in situ*.

In conclusion, we demonstrated that human and automated classification systems can achieve high sensitivity and specificity in discriminating images of AWM and nodal tissue acquired using FCM and extracellular fluorophores. We believe that the results of this study will facilitate clinical translation of FCM as an intraoperative imaging modality to reduce the incidence of conduction disturbances during surgical correction of congenital heart disease

## Supporting Information

S1 Appendix Supplemental methods. (PDF)

S1 Fig Anatomical overview of the right atrium from fixed rodent heart. The right atrium was labeled with wheat germ agglutinin (yellow) and antihyperpolarization-activated cyclic nucleotide-gated potassium channel 4 (green) to visualize the extracellular space and nodal cells, respectively. Outlined region (blue line) acquired using a 10x objective.
(TIF)

S1 Table Parameters for ROC analysis. (TIF)

S2 Table Comprehensive summary of human and automated tissue classification. (TIF)

## References

[1] Go AS, Mozaffarian D, Roger VL, Benjamin EJ, Berry JD, Blaha MJ, et al Heart disease and stroke statistics—2014 update: a report from the American Heart Association. Circulation. 2014 1 21;129(3):e28–e292. 10.1161/01.cir.0000441139.02102.80 24352519 PMC5408159

[2] Lillehei CW, Sellers RD, Bonnabeau RC, Eliot RS. Chronic Postsurgical Complete Heart Block. With Particular Reference to Prognosis, Management, and a New P-Wave Pacemaker. J Thorac Cardiovasc Surg. 1963 10;46:436–56. . 14074450

[3] Bonatti V, Agnetti A, Squarcia U. Early and late postoperative complete heart block in pediatric patients submitted to open-heart surgery for congenital heart disease. Pediatr Med Chir. 1998 May-Jun;20(3):181–6. . 9744009

[4] Weindling SN, Saul JP, Gamble WJ, Mayer JE Jr, Wessel D, Walsh EP. Duration of complete atrioventricular block after congenital heart disease surgery. The American Journal of Cardiology. 1998;82(4):525–7. 9723647 10.1016/s0002-9149(98)00375-0

[5] Bogers AJ, Head SJ, de Jong PL, Witsenburg M, Kappetein AP. Long term follow up after surgery in congenitally corrected transposition of the great arteries with a right ventricle in the systemic circulation. Journal of cardiothoracic surgery. 2010;5:74 Pubmed Central PMCID: 2954981. Epub 2010/10/06. eng. 10.1186/1749-8090-5-74 20920167 PMC2954981

[6] Graham TP Jr., Bernard YD, Mellen BG, Celermajer D, Baumgartner H, Cetta F, et al Long-term outcome in congenitally corrected transposition of the great arteries: a multi-institutional study. J Am Coll Cardiol. 2000 7;36(1):255–61. . Epub 2000/07/18. eng. 10898443 10.1016/s0735-1097(00)00682-3

[7] Anderson RH, Ho SY, Becker AE. The surgical anatomy of the conduction tissues. Thorax. 1983 6;38(6):408–20. . Pubmed Central PMCID: 459576. Epub 1983/06/01. eng. 6348996 10.1136/thx.38.6.408 PMC459576

[8] Dick M 2nd, Norwood WI, Chipman C, Castaneda AR. Intraoperative recording of specialized atrioventricular conduction tissue electrograms in 47 patients. Circulation. 1979 1;59(1):150–60. . 758107 10.1161/01.cir.59.1.150

[9] Marelli AJ, Mackie AS, Ionescu-Ittu R, Rahme E, Pilote L. Congenital heart disease in the general population: changing prevalence and age distribution. Circulation. 2007 1 16;115(2):163–72. . 17210844 10.1161/CIRCULATIONAHA.106.627224

[10] Lincoln C, Butler P, Logan-Sinclair R, Anderson RH. A cardiac conduction monitor and probe for intraoperative identification of conduction tissue. Br Heart J. 1979 9;42(3):339–44. . Pubmed Central PMCID: 482157. 508456 10.1136/hrt.42.3.339 PMC482157

[11] Sharma P, Meining AR, Coron E, Lightdale CJ, Wolfsen HC, Bansal A, et al Real-time increased detection of neoplastic tissue in Barrett's esophagus with probe-based confocal laser endomicroscopy: final results of an international multicenter, prospective, randomized, controlled trial. Gastrointest Endosc. 2011 9;74(3):465–72. Pubmed Central PMCID: 3629729. 10.1016/j.gie.2011.04.004 21741642 PMC3629729

[12] Thiberville L, Salaun M, Lachkar S, Dominique S, Moreno-Swirc S, Vever-Bizet C, et al Human in vivo fluorescence microimaging of the alveolar ducts and sacs during bronchoscopy. Eur Respir J. 2009 5;33(5):974–85. 10.1183/09031936.00083708 19213792

[13] Wu K, Liu JJ, Adams W, Sonn GA, Mach KE, Pan Y, et al Dynamic real-time microscopy of the urinary tract using confocal laser endomicroscopy. Urology. 2011 7;78(1):225–31. 10.1016/j.urology.2011.02.057 21601243 PMC4038103

[14] Huang C, Kaza AK, Hitchcock RW, Sachse FB. Identification of nodal tissue in the living heart using rapid scanning fiber-optics confocal microscopy and extracellular fluorophores. Circulation Cardiovascular imaging. 2013 9 1;6(5):739–46. 10.1161/CIRCIMAGING.112.000121 23811748 PMC3928358

[15] Huang C, Kaza AK, Hitchcock RW, Sachse FB. Local delivery of fluorescent dye for fiber-optics confocal microscopy of the living heart. Frontiers in physiology. 2014;5. 10.3389/fphys.2014.00367 PMC4174735 25309455

[16] Fletcher RH, Fletcher SW. Clinical Epidemiology: The Essentials: Lippincott Williams & Wilkins; 2005.

[17] Lasko TA, Bhagwat JG, Zou KH, Ohno-Machado L. The use of receiver operating characteristic curves in biomedical informatics. J Biomed Inform. 2005 10;38(5):404–15. . 16198999 10.1016/j.jbi.2005.02.008

[18] Bhavanandan VP, Katlic AW. The interaction of wheat germ agglutinin with sialoglycoproteins. The role of sialic acid. J Biol Chem. 1979 5 25;254(10):4000–8. . Epub 1979/05/25. eng. 108267

[19] Soderstrom KO. Lectin binding to collagen strands in histologic tissue sections. Histochemistry. 1987;87(6):557–60. . Epub 1987/01/01. eng. 2447040 10.1007/BF00492470

[20] Chandler NJ, Greener ID, Tellez JO, Inada S, Musa H, Molenaar P, et al Molecular architecture of the human sinus node: insights into the function of the cardiac pacemaker. Circulation. 2009 3 31;119(12):1562–75. Epub 2009/03/18. eng. 10.1161/CIRCULATIONAHA.108.804369 19289639

[21] Yoo S, Dobrzynski H, Fedorov VV, Xu SZ, Yamanushi TT, Jones SA, et al Localization of Na+ channel isoforms at the atrioventricular junction and atrioventricular node in the rat. Circulation. 2006 9 26;114(13):1360–71. . Epub 2006/09/13. eng. 16966585 10.1161/CIRCULATIONAHA.106.613182

[22] Lackey DP, Carruth ED, Lasher RA, Boenisch J, Sachse FB, Hitchcock RW. Three-dimensional modeling and quantitative analysis of gap junction distributions in cardiac tissue. Ann Biomed Eng. 2011 11;39(11):2683–94. Epub 2011/08/09. eng. 10.1007/s10439-011-0369-3 21822740

[23] Kwiterovich KA, Maguire MG, Murphy RP, Schachat AP, Bressler NM, Bressler SB, et al Frequency of adverse systemic reactions after fluorescein angiography. Results of a prospective study. Ophthalmology. 1991 7;98(7):1139–42. . 1891225 10.1016/s0161-6420(91)32165-1

[24] Kwan AS, Barry C, McAllister IL, Constable I. Fluorescein angiography and adverse drug reactions revisited: the Lions Eye experience. Clinical & experimental ophthalmology. 2006 Jan-Feb;34(1):33–8. . 16451256 10.1111/j.1442-9071.2006.01136.x

[25] Wallace MB, Meining A, Canto MI, Fockens P, Miehlke S, Roesch T, et al The safety of intravenous fluorescein for confocal laser endomicroscopy in the gastrointestinal tract. Aliment Pharmacol Ther. 2010 3;31(5):548–52. 10.1111/j.1365-2036.2009.04207.x 20002025

[26] Alford R, Simpson HM, Duberman J, Hill GC, Ogawa M, Regino C, et al Toxicity of organic fluorophores used in molecular imaging: literature review. Mol Imaging. 2009 Dec;8(6):341–54. . Epub 2009/12/17. eng. 20003892

[27] Camelliti P, McCulloch AD, Kohl P. Microstructured cocultures of cardiac myocytes and fibroblasts: a two-dimensional in vitro model of cardiac tissue. Microscopy and microanalysis: the official journal of Microscopy Society of America, Microbeam Analysis Society, Microscopical Society of Canada. 2005 6;11(3):249–59. . 16060978 10.1017/S1431927605050506

[28] Hong G, Lee JC, Robinson JT, Raaz U, Xie L, Huang NF, et al Multifunctional in vivo vascular imaging using near-infrared II fluorescence. Nat Med. 2012 12;18(12):1841–6. Pubmed Central PMCID: 3595196. 10.1038/nm.2995 23160236 PMC3595196

[29] Diaspro A. Confocal and two-photon microscopy: foundations, applications, and advances New York: Wiley-Liss; 2002.

[30] Yicong W, Li X. Two-photon Fluorescence Endomicroscopy. 2010. In: Advances in Lasers and Electro Optics [Internet]. InTech. Available: http://www.intechopen.com/articles/show/title/two-photon-fluorescence-endomicroscopy.

[31] Liu G, Chen Z. Fiber-based combined optical coherence and multiphoton endomicroscopy. J Biomed Opt. 2011 3;16(3):036010 Pubmed Central PMCID: 3188604. 10.1117/1.3555180 21456873 PMC3188604

[32] Wallace SJ, Morrison JL, Botting KJ, Kee TW. Second-harmonic generation and two-photon-excited autofluorescence microscopy of cardiomyocytes: quantification of cell volume and myosin filaments. J Biomed Opt. 2008 Nov-Dec;13(6):064018 10.1117/1.3027970 19123664

[33] Hoy CL, Durr NJ, Chen P, Piyawattanametha W, Ra H, Solgaard O, et al Miniaturized probe for femtosecond laser microsurgery and two-photon imaging. Optics express. 2008 6 23;16(13):9996–10005. . Pubmed Central PMCID: 3143712. 18575570 10.1364/oe.16.009996 PMC3143712

[34] Zhou H, Lange K, Suchard MA. Graphics Processing Units and High-Dimensional Optimization. Statistical science: a review journal of the Institute of Mathematical Statistics. 2010 8 1;25(3):311–24. . Pubmed Central PMCID: 3155776. 21847315 10.1214/10-STS336 PMC3155776

