# Supplementary material for: Sensitivity and Specificity of Cardiac Tissue Discrimination Using Fiber-Optics Confocal Microscopy
Source: PLoS One. 2016 Jan 25;11(1):e0147667. doi: 10.1371/journal.pone.0147667 (PMC4725960; doi:10.1371/journal.pone.0147667)
Supplement: S1 Appendix — (PDF) [file pone.0147667.s001.pdf]

Supplemental Methods

Tissue Preparations

Animal procedures were approved by the University of Utah Institutional Care and Use Committee and followed the guidelines of the National Institutes of Health Guide for the Care and Use of Laboratory Animals.

Rodent

Young adult Sprague-Dawley rats (≈300 g) were sacrificed with an anesthetic intraperitoneal injection of sodium pentobarbital (40 mg/kg) containing heparin (500 IU/kg). Following sacrifice, the hearts were rapidly excised and mounted on a Langendorff perfusion system [1]. Isolated hearts were then continuously perfused with oxygenated, high-K/low-Ca Tyrode's solution (in mmol/L: 92 NaCl, 11 dextrose, 13.2 KCl, 5 $MgCl_2$, 24 HEPES, 20 taurine, 5 creatine, 5 $C_3H_3NaO_3$, 1 NaH_2PO_4, 0.25 $CaCl_2$; pH 7.2; ≈10°C) at a flow rate of 10-15 mL/min. For fixed tissue preparations, perfusion was switched after 5 min to a perfusate of Tyrode's solution containing 4% paraformaldehyde (pH 7.4). Following a 10-min perfusion of fixative solution, the hearts were immersed in phosphate buffered saline (PBS) containing 4% paraformaldehyde for 24 h at 4°C. Tissue from the AWM, SAN, and AVN were dissected from the fixed hearts and stored in PBS at 4°C prior to immunofluorescent labeling.

Ovine

Hearts were obtained from a study on a preterm model of neonatal chronic lung disease [2]. Hearts and fixed tissue were prepared in a similar manner based on the previously described rodent model.

Human

Studies were reviewed and approved for exemption by the institutional review board at the University of Utah. We obtained fixed tissue samples from an explanted autopsy heart of a 38-week-old fetus stored at the Department of Pathology, Primary Children's Medical Center, Salt Lake City, Utah.

Fluorescent Labeling of Fixed Tissue

Fixed tissue preparations from rat, neonatal lamb, and human were washed 3x in PBS and incubated overnight with wheat germ agglutinin (WGA) conjugated to CF488A (29022-1; Biotium, Hayward, CA; 1:25). The next day, lamb and human tissue preparations were rinsed and stored in PBS until imaging. Rat tissue preparations were further labeled

using established methods [3]. In short, the preparations were rinsed 3x in PBS then permeabilized and blocked for 1 h in PBS solution containing 0.5% Triton X-100 and 4% normal goat serum (16210-064; Gibco, Grand Island, NY). Immediately following, the preparations were bathed for 1 h in Image-iT Signal Enhancer (I36933; Invitrogen, Carlsbad, CA). Preparations were then rinsed in PBS and incubated overnight on a laboratory platform rocker at room temperature with primary antibody solution. The primary antibody solution consisted of PBS, 4% normal goat serum, and antihyperpolarization-actived cyclic nucleotide-gated potassium channel 4 (HCN4; ab69054; Abcam, Cambridge, MA; 1:100). The following day, the preparations were washed 3x in PBS then incubated overnight on a rocker at room temperature in PBS solution containing secondary IgG (goat anti-rabbit IgG H+L) conjugated to Alexa Fluor 555 (A-21429; Invitrogen; 1:40). The next day, preparations were given a final rinse and stored in PBS until imaging.

Laser-scanning Confocal Imaging Protocol

Fluorescently labeled tissue preparations from rodent, neonatal lamb, and human were imaged using a conventional laser-scanning confocal microscope (Zeiss LSM5 Duo; Zeiss, Jena, Germany) based on established methods [3, 4]. Images of WGA-CF488A associated fluorescence were acquired using an Argon/2 ion laser for excitation at 488 nm and bandpass filter for 505 to 555 nm emissions. Images of anti-HCN4-Alexa 555 associated fluorescence were acquired using a HeNe laser for excitation at 543 nm and a long pass filter for >560 nm emissions. High magnification image stacks were captured using a 40x oil immersion lens having a numeric aperture of 1.3. These 3D image stacks were acquired at a 0.2x0.2x0.2 μm spatial resolution (*xyz* dimensions) with a *xy* field of view of 204.8x204.8 μm and a *z*-scan range of 50 μm. Anatomical overview images were acquired with the conventional confocal microscope using a 2.5x and 10x air objective with numeric apertures of 0.12 and 0.30, respectively.

Texture Analysis of Tissue Microstructure

The spatial regularity of the extracellular space in CCM, $FCM_{topical}$, and $FCM_{carrier}$ images was analyzed using two previously described methods of image texture analysis [3, 5-8]. For the first method, we applied a discrete 2-dimensional Fourier transform to decompose images into their spatial frequency components. From these transformed images, we sampled intensities within circular sectors bounded by spatial frequencies between 0.07 to 0.2 μm^{-1}. The intensities within the circular sectors were binned along central angles of 10° and normalized across all bins and local maxima. The

distribution of the binned intensities provides an indication of the spatial regularity of the extracellular space. Images composed of regions with regular striations exhibit a distribution of binned intensities with a pronounced maximum, while a flat distribution is observed in images composed of more heterogeneous content. Peaked and flat distributions of intensities indicate low and high spatial regularity, respectively.

The second method of texture analysis was based on second order image moments. Image moments provide a description of regions within an image in relation to a reference point or origin. Fundamental geometrical properties of regions can be extracted from image moments. In particular, we determined from second-order image moments the local orientation of structures found within regions of an image. Thus, we decomposed images into subregions and calculated the local orientation of these subregions based on second-order image moments. The relative frequency distribution of the local orientations within all subregions of an image was used to characterize the image's spatial regularity. Similar to the previously described Fourier analysis, Peaked and flat distribution of local orientations indicate low and high spatial regularity, respectively.

We defined a measure of spatial regularity, denoted as I_{15}, based on the shape of the intensity and local orientation distributions as calculated from both methods of texture analysis. The I_{15} value was calculated from the sum of intensities and local orientations within 15° of the peak of the angular spectra for all CCM, $FCM_{topical}$, and $FCM_{carrier}$ images.

References

1. Langendorff O. Untersuchungen am überlebenden Säugetierherzen. Pflügers Arch. 1895;61:291-332.
2. Rehan VK, Fong J, Lee R, Sakurai R, Wang ZM, Dahl MJ, et al. Mechanism of reduced lung injury by high-frequency nasal ventilation in a preterm lamb model of neonatal chronic lung disease. Pediatr Res. 2011 Nov;70(5):462-6. PubMed PMID: 21814155. Pubmed Central PMCID: 3189277.
3. Huang C, Kaza AK, Hitchcock RW, Sachse FB. Identification of nodal tissue in the living heart using rapid scanning fiber-optics confocal microscopy and extracellular fluorophores. Circulation Cardiovascular imaging. 2013 Sep 1;6(5):739-46. PubMed PMID: 23811748.
4. Lackey DP, Carruth ED, Lasher RA, Boenisch J, Sachse FB, Hitchcock RW. Three-dimensional modeling and quantitative analysis of gap junction distributions in cardiac tissue. Ann Biomed Eng. 2011 Nov;39(11):2683-94. PubMed PMID: 21822740. Epub 2011/08/09. eng.
5. Gonzalez RC, Woods RE. Digital Image Processing. Reading, MA: Addison-Wesley; 1992.

6. Lichter JG, Carruth E, Mitchell C, Barth AS, Aiba T, Kass DA, et al. Remodeling of the sarcomeric cytoskeleton in cardiac ventricular myocytes during heart failure and after cardiac resynchronization therapy. J Mol Cell Cardiol. 2014 Jul;72:186-95. PubMed PMID: 24657727. Pubmed Central PMCID: 4077200.

7. Sachse FB, Torres NS, Savio-Galimberti E, Aiba T, Kass DA, Tomaselli GF, et al. Subcellular Structures and Function of Myocytes Impaired During Heart Failure Are Restored by Cardiac Resynchronization Therapy. Circul Res. 2012 January 17, 2012.

8. Lasher RA, Pahnke AQ, Johnson JM, Sachse FB, Hitchcock RW. Electrical stimulation directs engineered cardiac tissue to an age-matched native phenotype. Journal of tissue engineering. 2012;3(1):2041731412455354. PubMed PMID: 22919458. Pubmed Central PMCID: 3424978.
