# Supplementary figures and images for: Sensitivity and Specificity of Cardiac Tissue Discrimination Using Fiber-Optics Confocal Microscopy
Source: PLoS One. 2016 Jan 25;11(1):e0147667. doi: 10.1371/journal.pone.0147667 (PMC4725960; doi:10.1371/journal.pone.0147667)

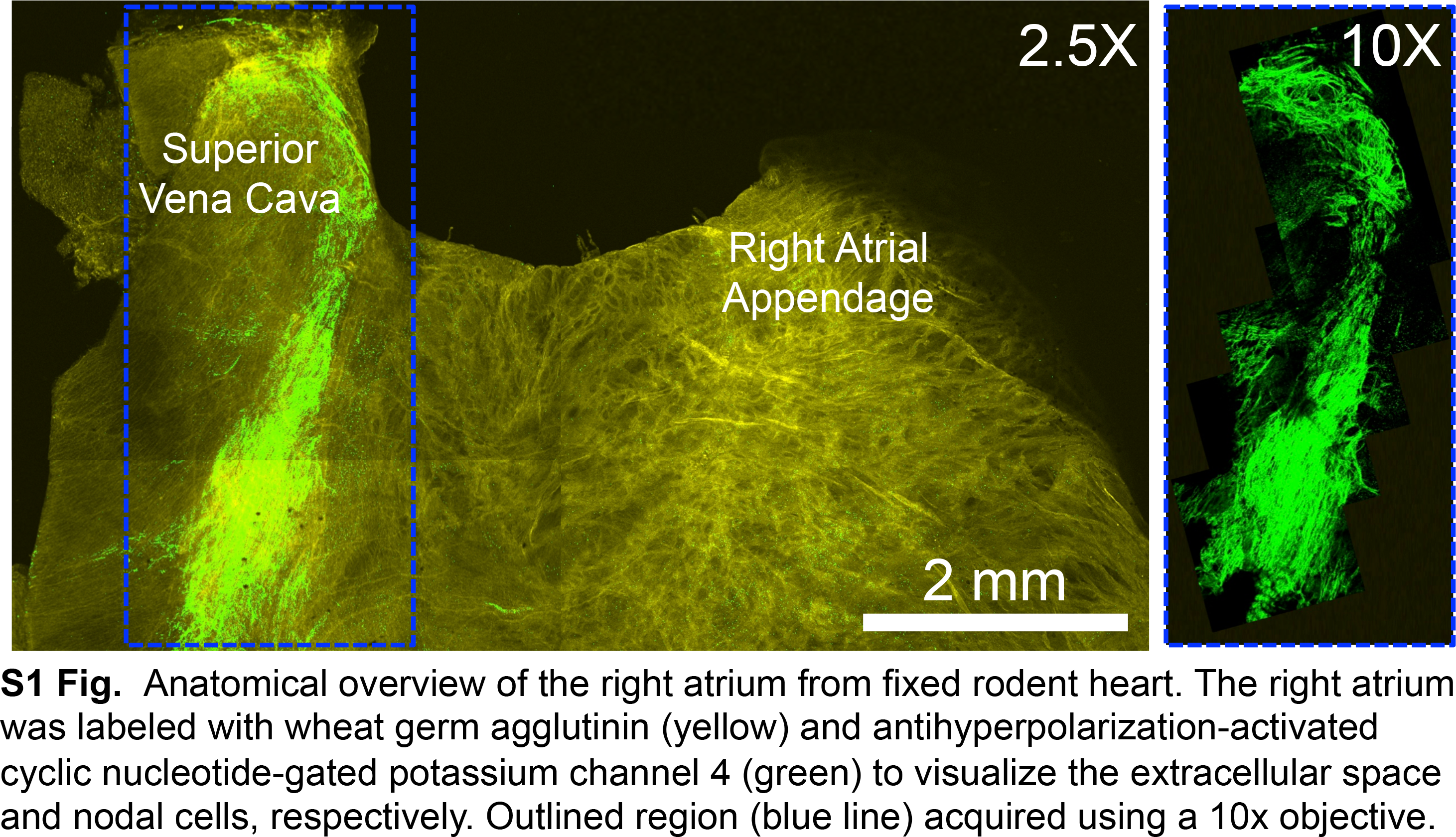

Supplement: S1 Fig — The right atrium was labeled with wheat germ agglutinin (yellow) and antihyperpolarization-activated cyclic nucleotide-gated potassium channel 4 (green) to visualize the extracellular space and nodal cells, respectively. Outlined region (blue line) acquired using a 10x objective. (TIF) [file pone.0147667.s002.tif]

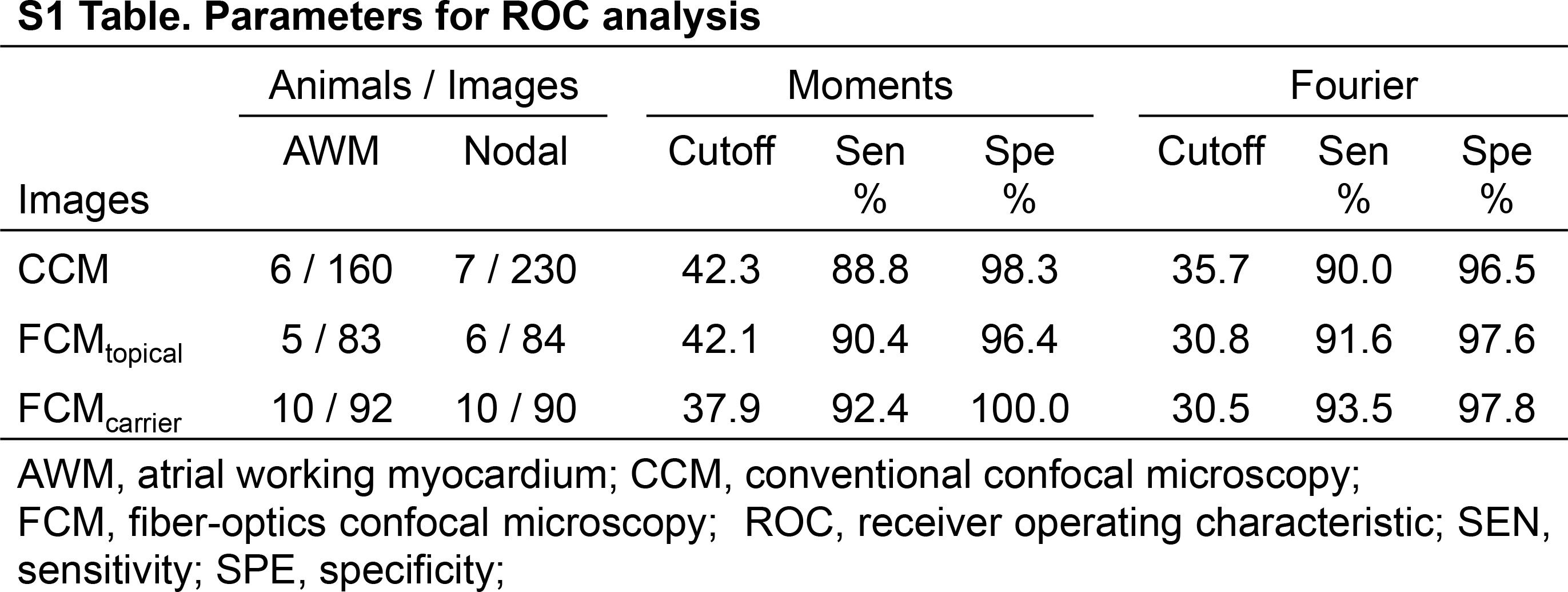

Supplement: S1 Table — (TIF) [file pone.0147667.s003.tif]

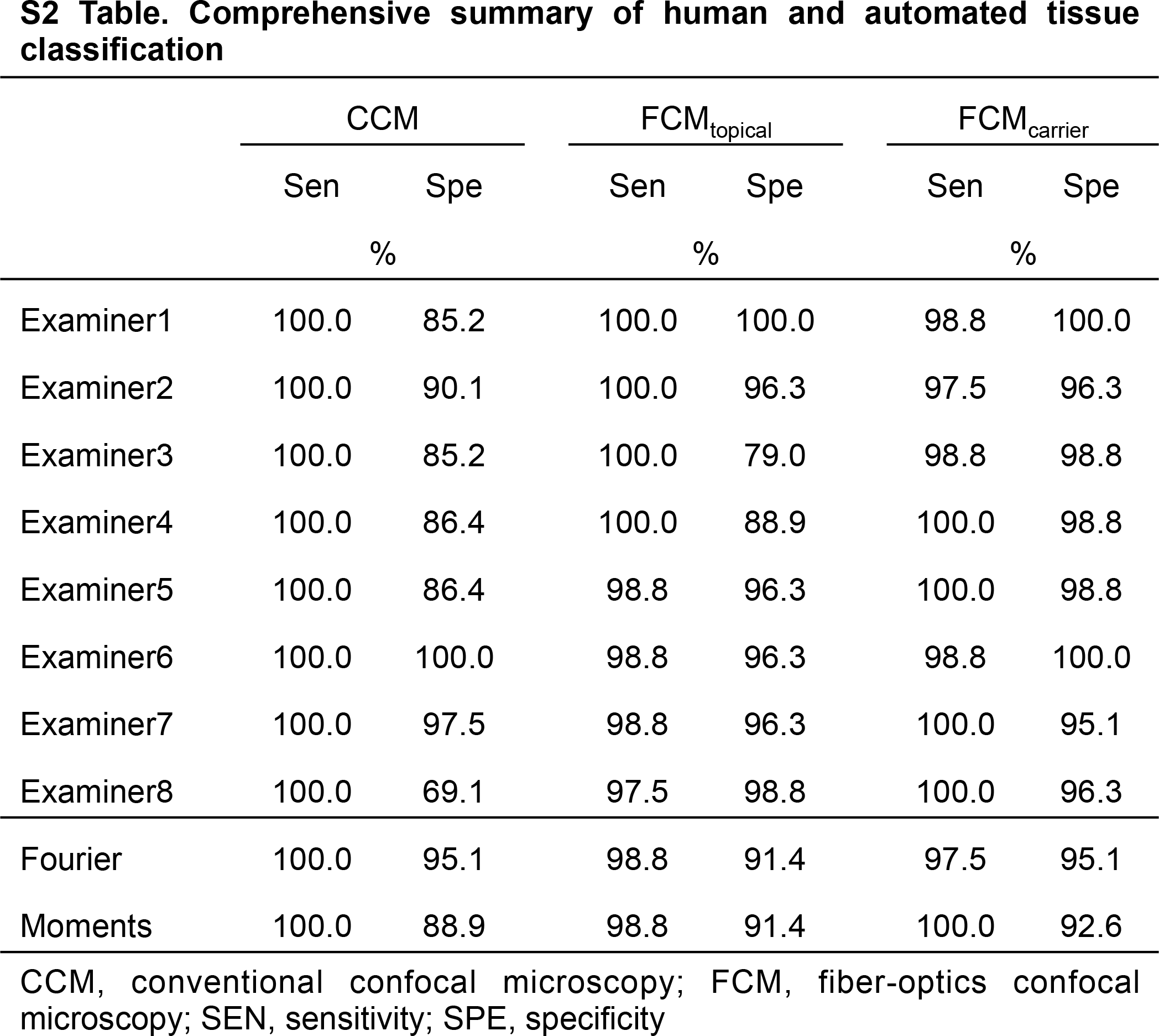

Supplement: S2 Table — (TIF) [file pone.0147667.s004.tif]
